# Semaphorin 6A Improves Functional Recovery in Conjunction with Motor Training after Cerebral Ischemia

**DOI:** 10.1371/journal.pone.0010737

**Published:** 2010-05-20

**Authors:** Andreas Rogalewski, Tanjew Dittgen, Matthias Klugmann, Friederike Kirsch, Carola Krüger, Claudia Pitzer, Jens Minnerup, Wolf-Rüdiger Schäbitz, Armin Schneider

**Affiliations:** 1 Sygnis Bioscience, Heidelberg, Germany; 2 Department of Neurology, University of Muenster, Muenster, Germany; 3 Institute for Physiological Chemistry and Pathobiochemistry, Johannes-Gutenberg-University Mainz, Mainz, Germany; Julius-Maximilians-Universität Würzburg, Germany

## Abstract

We have previously identified Semaphorin 6a (Sema6A) as an upregulated gene product in a gene expression screen in cortical ischemia [1]. Semaphorin 6a was regulated during the recovery phase following ischemia in the cortex. Semaphorin 6a is a member of the superfamily of semaphorins involved in axon guidance and other functions. We hypothesized that the upregulation indicates a crucial role of this molecule in post-stroke rewiring of the brain. Here we have tested this hypothesis by overexpressing semaphorin 6a in the cortex by microinjection of a modified AAV2-virus. A circumscribed cortical infarct was induced, and the recovery of rats monitored for up to 4 weeks using a well-established test battery (accelerated rotarod training paradigm, cylinder test, adhesive tape removal). We observed a significant improvement in post-ischemic recovery of animals injected with the semaphorin 6a virus versus animals treated with a control virus. We conclude that semaphorin 6a overexpressed in the cortex enhances recovery after cerebral ischemia. Semaphorin 6a may represent a novel therapeutic candidate for the treatment of chronic stroke.

## Introduction

Stroke is a major health problem in industrialized societies. Despite numerous attempts at developing acute stroke therapies aimed at minimizing acute infarct development, the only approved therapy so far is recombinant tissue plasminogen activator (rtPA). In recent years, the attention of the stroke community has therefore also put increased emphasis on understanding processes of post-stroke recovery, and their potential exploitability for therapeutic purposes.

The brain has a remarkable ability to adapt to changes after stroke. Mechanisms that contribute to this plasticity are re-mapping and expansion of cortical areas to neighboring regions of functional motor cortex areas after injury [2]–[4], and reorganization of ipsilateral cortical regions distant from the injury [5]. The correlates of these plastic changes are changes in neuronal networks, mediated by the generation of new neurons (neurogenesis) [6], [7], changes in dendritic and synaptic morphology [8], and changes in long-distance connectivity, requiring axonal outgrowth and pathfinding.

On a search for molecular determinants of those processes we have conducted a gene expression study in the photothrombotic model and searched for differentially expressed genes in the ipsi- and contralateral homotopic cortex [1]. One of the regulated genes appeared highly interesting in the context of post-stroke re-organization of neuronal networks after injury and axonal pathfinding to define new connections. This was a member of the semaphorin family of axonal pathfinding genes, Semaphorin 6A (Sema6A). This gene was induced ipsilaterally starting 48 h post ischemia, and increased in expression further at 21 d after ischemia (by quantitative PCR the regulation was determined as approximately 2-fold at 48 h, and 3-fold after 21 d in the ipsilateral cortex) [1]. At 21 d there was also significant upregulation detectable in the contralateral homotopic cortex (app. 2.5-fold). This induction on the mRNA level could be confirmed on the protein level by immunohistochemistry: At 48 h and 21 d, there was strong periinfarct expression of Semaphorin VIa in neurons, while induction on the contralateral homotopic cortex was only detected at 21 d. This regulation pattern suggested involvement in long-term plasticity processes in the periinfarct and homotopic contralateral cortex.

Semaphorins are involved in many processes in development, including cell migration, axon guidance, or dendritogenesis [9]. Transmembrane semaphorins of class 6 are important in vivo mediators of axon guidance and cell migration in different parts of the brain. Sema6A is characterized by an extracellular semaphorin domain, a transmembrane domain, and a long cytoplasmic domain [10]. There is evidence that these transmembrane semaphorins and their invertebrate orthologues can also function as receptors, also due to the presence of the long cytoplasmic tail harbouring an Evl domain. Sema6A can repel sympathetic and dorsal root ganglion axons in vitro [11] suggesting that it fulfills the functions of an axonal guidance signal.

Sema6A was identified in a gene-trap approach to find genes involved in axonal pathfinding [12]. The most striking phenotype of the Sema6A knock-out that was observed in this study was a defect in thalamocortical projections in homozygous k.o. animals [12]. In the cerebellum, Sema6A is involved in migration of granule cells [13]. Here, the receptor PlxA2 seems to be the responsible counterpart of Sema6A.

Recently, a striking defect in the building of the corticospinal tract (CST) has been described in Sema6A mutants [14]. This function seems to require the PlxnA4.

Here we have studied the effects of enhancing the expression of Sema6A in the recovery phase after cerebral cortical ischemia by AAV2-mediated gene transfer to the cortex.

## Materials and Methods

### Ischemic model and virus injection

24 Animals were anesthetized with an intraperitoneal injection of xylazine hydrochloride (Bayer, Leverkusen Germany) and ketamine hydrochloride (WDT, Garbsen, Germany). A PE-50 polyethylene tube was inserted into the right femoral artery for continuous monitoring of mean arterial blood pressure, and blood gases. During the experiment rectal temperature was monitored and maintained at 37°C by a thermostatically controlled heating pad (Föhr Medical Intruments, Germany).

Photothrombotic ischemia was induced in the rat parietal cortex [15]. Animals were placed in a stereotaxic frame, and the scalp was incised for exposure of the skull surface. For illumination, a fiber-optic bundle with a 1.5-mm aperture was placed stereotaxically onto the skull 0.5 mm ventral to the bregma and 4 mm lateral from the midline on the right side. The skull was illuminated with a cold, white light beam (150 W) for 25 minutes. During the first 2 minutes of illumination, the dye rose bengal (0.133 mL/kg body weight, 10 mg/mL saline) was injected intravenously. After surgery, the catheters were removed, and the animals were allowed to recover from the anesthesia and given food and water ad libitum.

The day following induction of cerebral ischemia virus was injected stereotactically at 3 positions in the ipsi- and contralateral hemisphere. The following coordinates were used: 3 mm lateral and 3.5 mm ventral to the bregma; 5.5 mm lateral and 0.5 mm ventral to the bregma and 4 mm lateral and 3.5 mm dorsal to the bregma. Injections were done by an experimenter blinded to the identity of the virus.

### AAV vector production

HEK 293 cells are plated 24 hours before transfection in complete DMEM. About 70% confluent cells are transfected with 12.5 µg AAV plasmid, 25 µg pFdelta6, 6.25 µg pRV1, 6.25 pH21, 330 µl 2.5 M CaCl_2_ and 2.4 ml H_2_O per 15 cm dish as follows(Klugmann, et al., 2005): The transfection mix is filtered through a 0.2 µm syringe filter. Under vigorous vortexing 13 ml of 2×HeBs buffer is added and precipitate is allowed to form for 2 min. 5 ml of the transfection solution is gently added to each plate dropwise. 16 hours after transfection, the medium is removed and replaced with 25 ml of fresh DMEM. 60–65 hours after transfection cells are gently washed with 25 ml warm 1× PBS and harvested in 25 ml 1×PBS using a cell scraper. Cells are pelleted at 800 g for 10 min, resuspended in 10 ml 150 mM NaCl, 20 mM Tris pH 8.0. Sodium deoxycholate (Sigma #D5670) is added to a final concentration of 0.5% and Benzonase endonuclease (Sigma #E1014) to a final concentration of 50 U/ml. Solution is mixed thoroughly, incubated at 37°C for 1 h. Cell debris is removed by centrifugation at 3000g×15min, 4°C. Heparin columns (1 ml HiTrap Heparin columns, Sigma #5-4836) are pre-equilibrated with 10 ml 150 mM NaCl, 20 mM Tris pH 8.0. The column is loaded at 1 ml/min. The column is washed with 20 ml 100 mM NaCl, 20 mM Tris pH 8.0 at 1 ml/min, 1 ml 200 mM NaCl, 20 mM Tris pH 8.0 and 1×1 ml 300 mM NaCl, 20 mM Tris pH 8.0. The virus is eluted in 1.5 ml 400 mM NaCl, 20 mM Tris pH 8.0, 3 ml 450 mM NaCl, 20 mM Tris pH 8.0 and 1.5 ml 500 mM NaCl, 20 mM Tris pH 8.0. The virus containing eluate is concentrated using self-contained AMICON ULTRA- 4 (100000 MWCO; Millipore; CatNo. UFC810024) filters at 2000 g for 2 min. Afterwards it is sterilized by filtration through a 13 mm 0.2 µm syringe filter. 10 µl purified vector are analysed on a Coomassie protein gel for purity.

### Genomic Titering of AAV

Viral DNA is extracted by diluting 2 µl of virus stock in 10 µl 10× ABI buffer (500 mM KCl, 100 mM Tris pH 8.0, 50 mM MgCl_2_) and 86 µl sterile water. 1 µl DNAseI is added and incubated at 37°C for 30 min. DNAse is inactivated at 70°C for 10 min. 10 µg of Proteinase K are added and the mixture is incubated for 1 hour at 50°C. Proteinase K is inactivated at 95°C for 20 min. Viral DNA is diluted 1∶50 in PCR grade water. The titer of the virus is determined by quantitavie PCR (Lightcycler, Roche Diagnostics) in comparison to plasmids with known dilutions. The following primers are used: WPRE for: GGC TGT TGG GCA CTG ACA AT; WPRE rev: CCG AAG GGA CGT AGC AGA AG; CBA titre for: TAT CAT ATG CCA AGT ACG CCC C; CBA titre rev: GGG CCA TTT ACC GTC ATT GA.

### RT-PCR

RNA of brains was isolated using the acidic phenol extraction protocol followed by QIAGEN RNeasy Mini Kit purification according to the manufacturer's recommendations. cDNA was synthesized from 5 µg total RNA using oligo-dT primers and Superscript II Reverse Transcriptase (Invitrogen Corp.).

For the detection of the AAV-3x FLAG-semaphorin 6A a nested PCR strategy was pursued using the following primers: semaphorin_788_s (GAC TGA CCG CGT TAC TCC CAC AGG TGA) and semaphorin_1510_as (TCG AGC AGC AAT GTA GAG GGT TCT GTTC) for the first PCR giving a product of 722 bp (56°C annealing, 35 cycles). The second PCR primer pair - semaphorin_3X_Flag_1154_sense (ACC ATG GAC TAC AAA GAC CAT GAC GG) and semaphorin_1377_as (ATA CTG ATC GGC TCA GAA TCT TCT GGG) - generated the final PCR product of 223 bp (56°C annealing, 35 cycles).

### Behavioral Testing

All animals were operated on and tested in parallel (1 animal per group at once). In all animals, behavioral tests were performed before (baseline) and 3, 17, 24, and 31 days after ischemia by a blinded investigator. For Rotarod tests, the animals were not trained before ischemia. From day 6 onward rats were trained for a total of 10 days, with 2 days break in between, in an accelerated Rotarod training paradigm [16]. Rats were placed on an accelerating Rotarod cylinder, and the time the animals remained on the Rotarod was measured. Speed was increased from 4 to 40 rpm within 5 minutes. The trial ended if the animal fell off the rungs or gripped the device and spun around for 2 consecutive revolutions without attempting to walk on the rungs. An arbitrary time limit of 500 seconds was set for the rats on the Rotarod cylinder in training and in the testing procedures. Every training day the rats received 10 training sessions.

The adhesive removal test was done both before (baseline) and 3, 20, and 34 days after ischemia. Initially, 2 pieces of adhesive-backed paper dots (113.1 mm^2^) were used as bilateral tactile stimuli on the dorsal paw of each forelimb. The time to remove each stimulus from the forelimbs was recorded 3 trials per day for each forepaw. Individual trials were separated by 5 minutes. Before surgery, animals were trained for 3 days.

The cylinder test was done both before (baseline) and 3, 20, and 34 days after ischemia. The animals were not trained before ischemia. The rats were placed in a transparent plexiglas cylinder (20cm high, 20cm diameter) placed on a glass table for 5 minutes and recorded on video. For analysis, the number of independent placements of the forelimbs was measured over a time period of 30 seconds.

### Experimental setup

All animal experiments followed ethical standards, and protocols were approved by the respective government authorities. Male Wistar rats (Charles River; 280 to 320 g) were randomly assigned to groups with end points at day 31. Virus was injected 1 day following photothrombotic ischemia. Animals (n = 12 per group) were injected with either verum or vehicle virus by an operator blinded to the treatment.

### Statistics

Experiments were performed in a completely randomized and blinded manner. The experimenter was blinded to the identity of treatment at the time of the injection, and during the behavioural evaluation. Statistical analyses were done using JMP 8.01 (SAS Institute). A p value<0.05 was considered significant.

## Results

### Experimental setup

**Figure 1:**
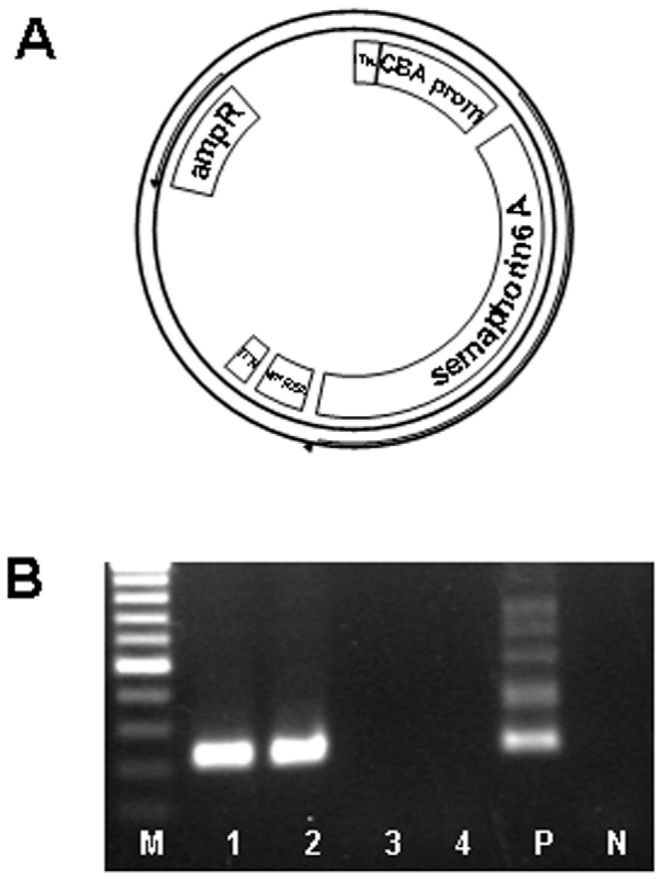
Map of the Sema6A-AAV2 construct, and persistence of expression. (A) The N-terminally 3xFLAG-tagged rat Sema6A was cloned behind the CMV/chicken beta actin promoter. (B) PCR for semaphorin-FLAG mRNA in the brain 35 days post injection of the virus. Shown is an agarose gel electrophoresis for amplimers specific for virus-originated Sema6a mRNA 35 days post injection in brain hemispheres. For the detection of AAV-3x FLAG-Semaphorin 6A nested PCR primers were used giving a final PCR product of 223 bp. The marker is a 100 bp-ladder. lane 1: (animal #22; Sema6A AAV2); lane 2: (animal #24; Sema6A AAV2); lane 3: (animal #8; empty AAV2); lane 4: (animal #11; empty AAV2); lane 5: positive control (AAV-3x FLAG-Semaphorin 6A plasmid); lane 6: negative control (water).

As expression tool for the semaphorin 6A protein we used the adeno-associated virus serotype 2 (AAV 2). We chose this virus because of its excellent infection of neurons, the relative longevity of expression, the safety of the virus, and the potential applicability as a human therapeutic vector [17]. This vector has been used in a large number of studies addressing neurobiological questions [18]–[22]. We cloned the rat semaphorin 6A open reading frame (N-terminally 3X FLAG-tagged) after the cytomegalovirus enhancer/chicken β-actin promoter chicken beta actin (CBA) promoter (Figure 1A). As control vectors, we packaged the AAV2-plasmid without any coding sequence (AAV2-empty).

Injection of the virus resulted in expression of Semaphorin 6A at least until the end of the experiment when we could detect virus-originated Semaphorin 6A mRNA in the verum- but not in the control-virus injected brains (31 days post injection; Figure 1B, compare lanes 1 and 2 versus 3 and 4).

**Figure 2:**
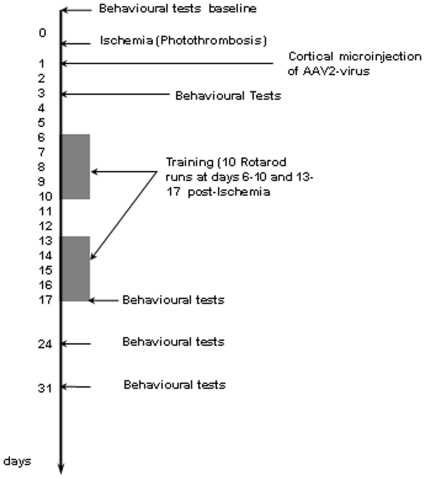
Schedule of the experimental setup. Photothrombotic ischemia was induced in the rat parietal cortex at day 0. 1 day after induction of ischemia, AAV2 was injected at 3 positions both on the ipsi- and contralateral hemisphere. Adhesive tape removal and cylinder tests were done at day 3, day 20, 27 and 34. Rotarod training was performed at day 6 to 10, day 13 to 17, day 24, and day 31.

Photothrombotic ischemia was performed using intravenous injection of bengal rose, and illumination of the cortical sensorimotor frontpaw area with a laser (coordinates 4 mm lateral and 0.5 mm ventral to the bregma). Semaphorin 6A AAV2 virus or control virus was injected bilaterally 1 day following photothrombotic ischemia at 3 sites per hemisphere (coordinates: 3 mm lateral and 3.5 mm ventral to the bregma; 5.5 mm lateral and 0.5 mm ventral to the bregma; 4 mm lateral and 3.5 mm dorsal to the bregma). These injection sites were targeted to the periischemic region. 12 animals per group were injected by an operator blinded to the virus identity. From day 6 onward rats were trained for a total of 10 days in an accelerated rotarod training paradigm, with 2 days break in between. Every training day the rats received 10 training sessions. The experimental setup scheme is given in figure 2.

### Semaphorin 6A overexpression improves poststroke motor recovery

**Figure 3:**
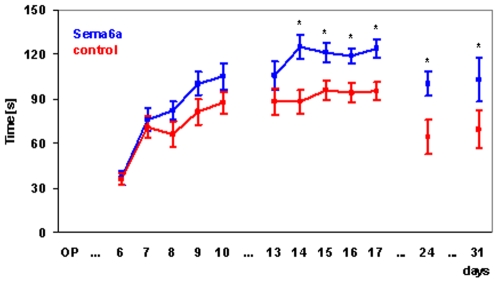
AAV2-mediated semaphorin 6a overexpression improves recovery over time as measured by Rotarod performance. Rats were placed on an accelerating Rotarod cylinder, and the time the animals remained on the Rotarod was measured. From day 6 onward rats were trained for a total of 10 days, with 2 days break in between, in an accelerated rotarod training paradigm. Every training day the rats received 10 training sessions. Animals that had received the Sema6a AAV2 virus (blue line) performed significantly better over time than animals that had received empty virus (red line) (repeated measures ANOVA, p<0.05). Post hoc tests revealed significant differences between groups from day 14 onward (p<0.05).

Animals were tested on an accelerating Rotarod at post-operative days 6 to 10, 13 to 17, 24, and 31 (Figure 3). Overall behavior of groups was different as judged by repeated measures ANOVA (p<0.05). Post-hoc tests reveal significant differences between groups starting at day 14, and staying significant thereafter.

**Figure 4:**
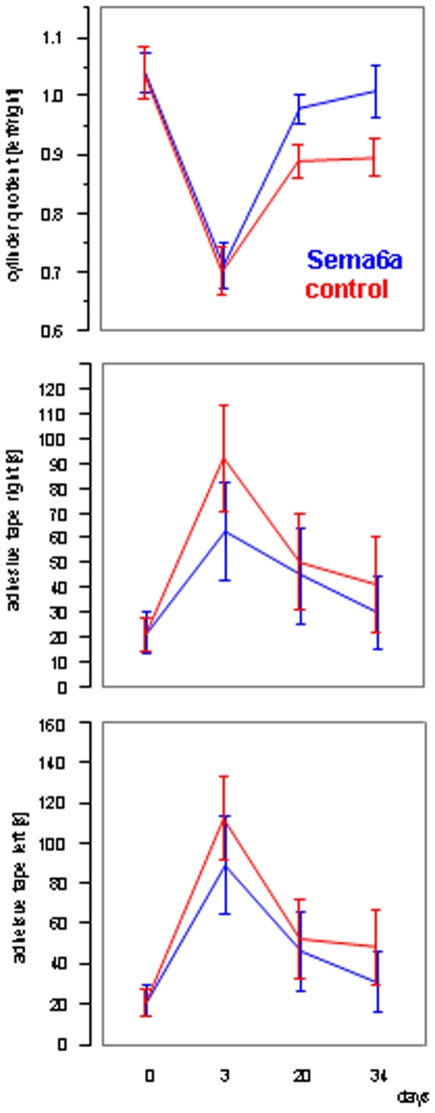
Effects of the cylinder test and adhesive tape removal test. (A) Cylinder test. (B) Adhesive removal task left. (C) Adhesive removal task left. The adhesive removal and cylinder tests were done both before (baseline) and 3, 20, and 34 days after ischemia. There was a strong trend towards better performance of the Sema6A AAV2 injected animals (blue line) in the cylinder test (repeated measures ANOVA, p = 0.0752). Although the mean performance of Sema6A AAV2 injected rats in the adhesive tape removal appears better than control animals, this difference was not significant.

In the cylinder test we observed a strong trend towards better performance of the group injected with the semaphorin 6a expressing virus (repeated measures ANOVA, p = 0.0752). This trend was increasing with time from the operation (Figure 4A).

Performance in the adhesive tape removal test for the left or right paw was not significantly different; however, the graph suggests a trend to better performance starting day 3 post surgery (Figure 4B and C).

In conclusion, Semaphorin6A overexpression led to a significant and strong improvement in Rotarod performance, and to a strong trend improvement in the cylinder test.

## Discussion

Axonal pathfinding is an integral prerequisite for rewiring neuronal networks that adapt to changed functional requirements. The most well-known family of proteins involved in axonal pathfinding are the semaphorins, that fall into eight classes of membraneous or secreted proteins sharing a 500 amino acid so-called sema domain, and guide growth cones by attraction or repulsion (for review see [23], [24]).

The only protein of the semaphorin class so far associated with cerebral ischemia is Semaphorin 3A, which was reported to be temporarily upregulated after MCAO [25], and induced after 14 d in the perilesional area of an infarct in the barrel cortex together with its receptor neuropilin I [26]. Complicating potential functions in post-stroke axonal pathfinding, Semaphorin 3A in the adult brain is also clearly associated with neuronal apoptosis [27].

Here we have defined a beneficial role of overexpression of the semaphorin Sema6A in the recovery phase after cortical ischemia. Together with physical training we observed positive effects on functional outcome. The clearest and significant effect was seen on Rotarod performance over time. It may be that the power of the Rotarod measurements was considerably higher compared to the adhesive tape removal or cylinder test. Alternatively, the effect may be strongest here since the physical training was also done on the Rotarod, thus providing a very specific training for this function. This may mean that the effect of Sema6A on plasticity is strongest when the most concomitant activity training is done.

At present it is unclear what properties of Sema6A contribute to the pro-recovery effect seen. One obvious possibility would be that rewiring of the post-stroke brain is enhanced. It is however also possible that Sema6a is somehow involved in targeting or migration of neuronal stem cells activated by the ischemic event.

These questions put aside, we have defined here a novel player in post-stroke recovery processes that is upregulated after ischemia over a long time frame, and whose overexpression enhances functional recovery.
